# Safety of intraoperative autologous plasma incubation of corneal grafts for reducing endothelial cell loss: a pilot study

**DOI:** 10.3389/fmed.2024.1368117

**Published:** 2024-08-12

**Authors:** Carolina Mercado, Cesar Hernández, Carolina Lopez-Rojas, Borja De La Sen-Corcuera, Eduardo Anitua, José Ignacio Barraquer, Angela Gutiérrez, Ernesto Otero, Alfonso L. Sabater

**Affiliations:** ^1^Escuela Superior de Oftalmología, Instituto Barraquer de América, Bogota, Colombia; ^2^Department of Ophthalmology, Bascom Palmer Eye Institute, University of Miami Miller School of Medicine, Miami, FL, United States; ^3^Institute for Regenerative Medicine and Oral Implantology, Vitoria, Spain; ^4^Biotechnology Institute (BTI), Vitoria-Gasteiz, Spain

**Keywords:** plasma rich in growth factors, penetrating keratoplasty, cornea transplantation, corneal endothelium, activated platelet rich plasma

## Abstract

**Background/aims:**

Corneal endothelial cell loss contributes to transplant failure. Autologous plasma products (APP) activate salvaging pathways that can prevent oxidative stress perioperatively. This study aimed to evaluate the safety of intraoperative incubation of full-thickness corneal grafts in platelet-rich plasma (aPRP) and plasma rich in growth factors (PRGF-Endoret) in mitigating postoperative corneal endothelial cell loss (ECL).

**Methods:**

Pilot study including patients undergoing penetrating keratoplasty (PK) for various indications between June 2021 and December 2022. Patients were randomly assigned to receive either aPRP or PRGF-Endoret incubation, while those who declined intervention served as the control group. Demographic and clinical data were collected, including preoperative and postoperative endothelial cell count, intraocular pressure, pachymetry, and adverse reactions.

**Results:**

Thirty individuals who underwent PK completed follow-up: eight from the aPRP group, 10 from the PRGF-Endoret group, and 12 from the control group. No adverse events related to APP treatment were recorded. In the first and third postoperative months, the APP group had significantly lower ECL percentages (37% vs. 25%, *p* = 0.02, and 44% vs. 33%, *p* = 0.02, respectively); this trend was maintained in the sixth month. When stratified, the PRGF-Endoret group showed significant differences in ECL reduction compared to controls at both time points (*p* = 0.03 and *p* = 0.05, respectively). The aPRP group showed a similar statistically significant outcome exclusively on the third postoperative month (*p* = 0.04). APP tended to reduce corneal edema faster than controls. Hexagonality was significantly better in the APP groups in the first and third months, particularly in the PRGF-Endoret group (*p* < 0.005).

**Conclusion:**

Preoperative incubation with APP is safe and promotes better endothelial cell quality and quantity in the early postoperative period following PK. These findings suggest a potential clinical benefit in enhancing graft outcomes and warrant further investigation.

## Introduction

Corneal transplantation is a necessary procedure to restore sight for many ocular pathologies. Currently, it is the most common type of transplantation in the world (1). Like any other type of transplantation, it also carries many risks, including rejection, failure, and infection. Full-thickness corneal transplants, known as penetrating keratoplasty (PK), have a higher risk of failure than partial thickness or endothelial keratoplasty (EK) (2). Although EKs are currently preferred over full-thickness transplants, PKs are indicated in particular situations and thus are still frequently used (2). Due to its avascularity, the human cornea has an immunological advantage compared to other types of tissue by not requiring human leukocyte antigen (HLA)-crossmatch, but surgical manipulation of the tissue, tissue preservation time, and some recipient factors can lead to primary graft failure in up to 12–56% of PKs (3). According to a 2016 survey, only one tissue is available for every 70 diseased eyes (4); given the limited amount of tissue available for transplant, it is logical to focus on options to prevent grafts from failing so that patients do not have to undergo multiple transplants. The risk of failing depends on the longevity of corneal endothelial cells (CECs), which maintain the cornea’s adequate function and provide transparency.

Oxidative stress has been described as an important mechanism that leads to corneal endothelial cell loss (ECL) (5) in Fuchs endothelial dystrophy (6) and phacoemulsification (7). ECL occurs after surgical manipulation; for example, an immediate 25% ECL after surgery was observed in a cadaveric model (8). Several studies have followed ECL closely in the first months after optical PK and found that cell count decreases about 28–40% in the first 6 months and up to 50% in the first year, and continues to decrease slowly over the subsequent 4–10 years (9, 10). Recently, it has been found that although factors like death to preservation time and preoperative cell density do not influence transplant failure (11, 12), postoperative endothelial cell counts lower than 2,500 cells/mm^2^ correlate with decreased transplant longevity (13). Currently, the mainstay in corneal transplantation to reduce inflammation and risk of rejection is postoperative topical steroids; by controlling inflammation, there is less cytokine-induced endothelial cell stress (14).

Autologous blood products, while previously used in the ocular surface (15, 16) and retina (17), have never been used for endothelial cell protection and regeneration. Activated plasma rich in platelets (aPRP) and plasma rich in growth factors (PRGF-Endoret) are interchangeable in augmenting the platelet content and potentiating its regenerative effect (15). Their main difference lies in the standardized preparation of PRGF-Endoret, which ensures a controlled activation process and lower inflammatory cytokine levels by avoiding the white blood cell column (18, 19). Autologous plasma products (APP) contain multiple growth factors, such as tissue growth factor beta (TGF-β), fibroblast growth factor (FGF), platelet-derived growth factor-BB (PDGF-BB), and vascular endothelial growth factor (VEGF) (20), which scavenge free radicals released during oxidative stress and thus activate salvaging pathways that protect the corneal endothelium (21, 22). Our study aimed to determine the safety of a short incubation of full-thickness corneal grafts with aPRP and PRGF-Endoret therapy before PK to prevent the loss of endothelial cells.

## Materials and methods

**Figure 1:**
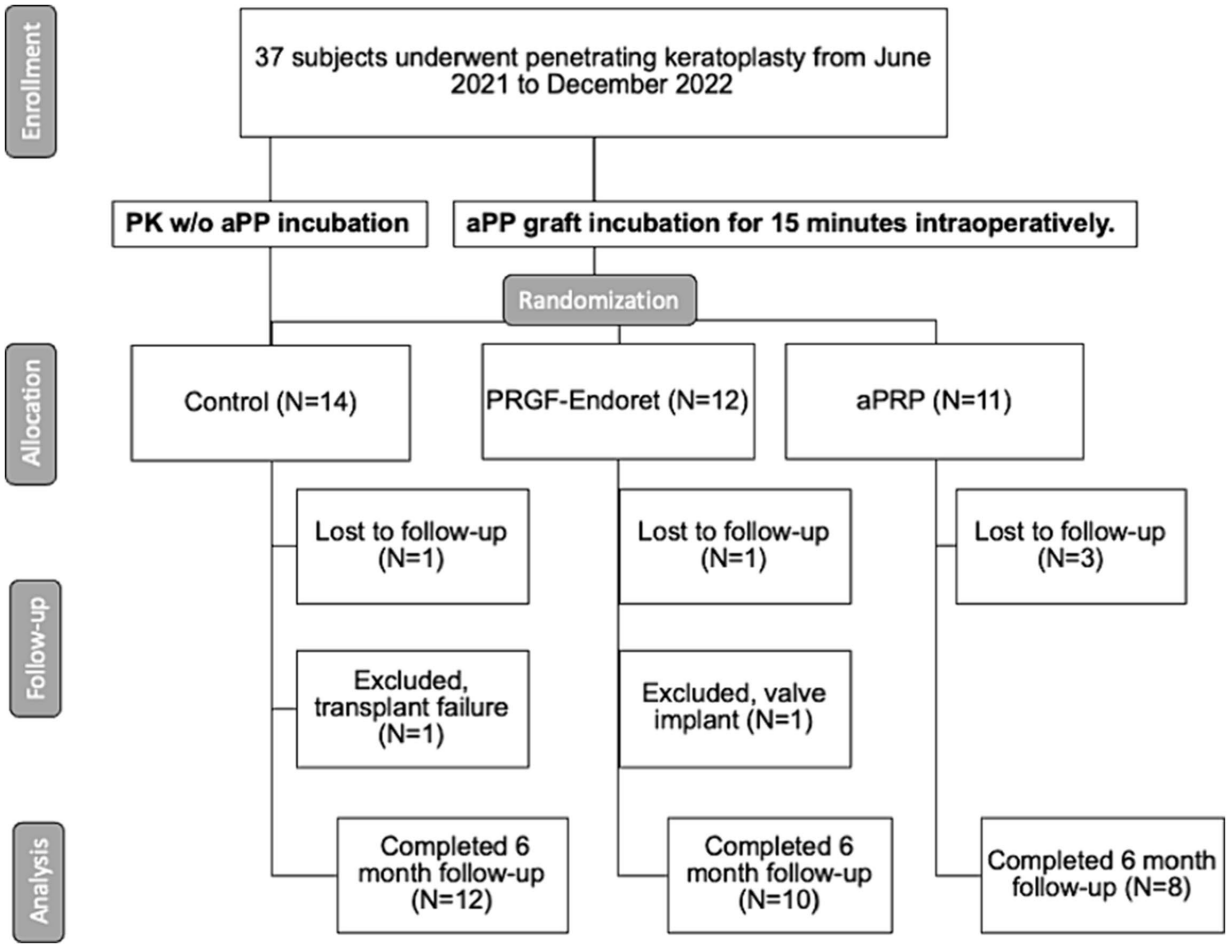
Subject disposition flow-chart: information regarding subject disposition during the trial is presented according to CONSORT guidelines. PK, penetrating keratoplasty; aPP, autologous plasma product; w/o, without; PRGF-Endoret, Plasma rich in growth factors; aPRP, activated platelet-rich plasma.

This pilot study was approved by the Escuela Superior de Oftalmología- Instituto Barraquer de América Ethics Committee (approval #27921992) and was conducted under the principles of the Declaration of Helsinki. We adhered to the CONSORT reporting guidelines for clinical trials, and this trial was registered in the ISRCTN registry (Trial number: ISRCTN12328428). Written informed consent was obtained from all study participants. Patients seen at Clínica Barraquer de América with an indication for PK based on clinical characteristics were included in the study. Patients underwent ophthalmological examinations by one of three different surgeons (EO, AG, JIB) in the Department of Cornea and External Diseases and agreed to undergo corneal transplantation. We included patients older than 18 years who were undergoing PK for any reason between June 2021 and December 2022 and were being treated by one of the previously mentioned surgeons. Exclusion criteria included patients from vulnerable populations (pregnant women, prisoners, intellectually, educationally, or economically disadvantaged individuals) or patients with previous trabeculectomies or valve implants. Patients with renal failure, anemia, or immunosuppression were also excluded because APP would not have the same effect. All the tissue was provided by the same Eye Bank (COBANCOL), with no more than 14 days of preservation. Tissue was excluded if it came from another institution or patients operated by other surgeons. Participants who agreed to undergo intervention were randomly assigned by block randomization to either of the treatment groups (aPRP incubation for 15 min intraoperatively or PRGF incubation for 15 min intraoperatively). Patients who declined intervention were followed as controls. The subject disposition chart is outlined in detail in Figure 1.

Informed consent was taken from all patients. On the morning of the procedure, patients of each intervention group had blood extracted by a skilled nurse, and six 4.5 mL citrated tubes were gathered. PRGF-Endoret was prepared according to the manufacturer’s instructions (PRGF-Endoret, BTI Biotechnology, Spain). In the aPRP group, the citrated tubes were centrifuged at 1700 rpm (460 g) for 10 min. The plasma was carefully isolated from the remaining white and red blood fractions and transferred to a 50 mL Falcon tube. Fifty microliters of calcium chloride 10% were added per milliliter of plasma collected, followed by a 45-min incubation at 37 degrees Celsius. As soon as incubation was complete, the plasma was refrigerated in the operating room until the procedure.

**Figure 2:**
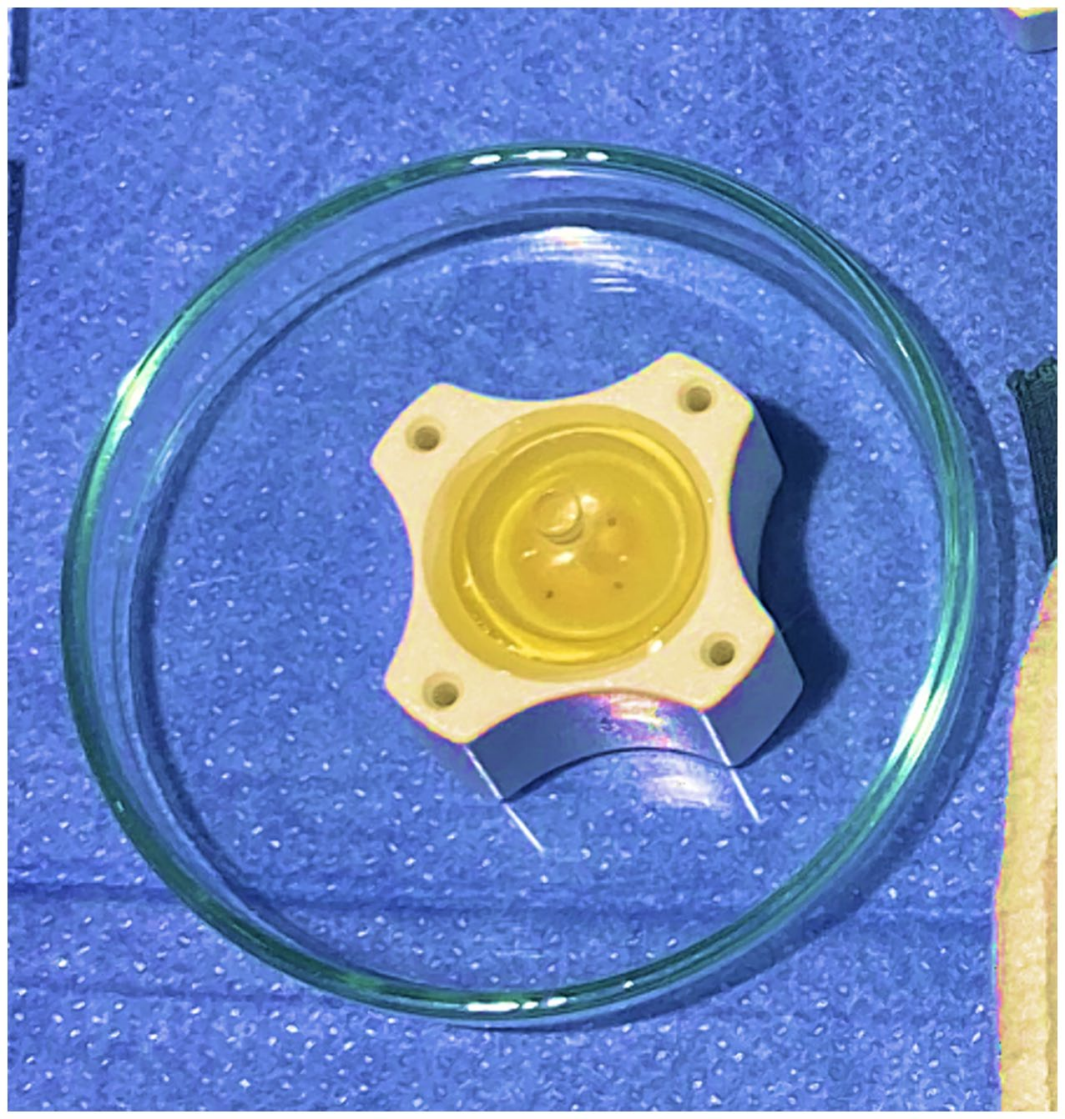
Intraoperative 15 minute room temperature incubation of full-thickness corneal graft in autologous blood product.

After the donor grafts for patients in the PRGF-Endoret and aPRP groups were trephined and ready for transplantation, a 15-min, room-temperature incubation of the grafts (Figure 2) was done by coating the corneal endothelium with the autologous blood products. At the same time, the surgeon prepared the recipient site. The blood products were not washed before positioning the corneal button. Patients in the control group underwent regular PK procedures without pre-incubation. Sixteen separate intrastromal sutures were placed, and a postoperative regimen of corticosteroids (prednisolone 1% six times daily for 14 days, tapered to four times daily for 14 days, then tapered to twice daily and continued for five months) and antibiotics (Ciprofloxacin 4 times daily for 14 days) was followed by every subject.

Patient characteristics such as age, sex, race, comorbidities, and history of previous corneal transplant were recorded. Preoperative and postoperative endothelial cell counts (CellChek XL, Konan Medical) were recorded in the first, third, and sixth month, along with intraocular pressures, pachymetry, and adverse reactions. Only patients who completed the 6-month study duration and did not have graft failure or trabeculectomies were included in the final analysis. Thirty-seven patients underwent PKs from June 2021 to December 2022 by the three surgeons in the study. Five patients were excluded because of loss of follow-up, one patient was excluded because he required a valve implant in the same procedure, and one had transplant failure.

This study aimed to prove the safety of aPRP and PRGF-Endoret in preventing ECL in full-thickness transplants. The primary outcome measure was to compare the ECL of patients who had preoperative aPRP or PRGF-Endoret incubation with patients who underwent regular PKs as proof of the effect of these autologous plasma products in the prevention of perioperative ECL, and to assess the safety of this therapy by reporting any abnormal reaction or adverse effects. As PRP and PRGF-Endoret are blood products with similar characteristics, we also analyzed their data as a whole in what is designated as the APP group.

Statistical analysis was performed using the Statistical Package for the Social Sciences 28.0 (SPSS Inc., Chicago, IL). Frequencies of demographic and clinical variables were calculated for each group. We assessed the normality of the data distribution using the Shapiro–Wilk test. Group means were compared using t-tests with a 95% confidence interval if the data was normally distributed. In cases where data did not follow a normal distribution, non-parametric tests such as the Mann–Whitney U test were used for comparisons.

## Results

### Demographics

Thirty individuals were included in this study. Eight patients were included in the aPRP group, 10 in the PRGF-Endoret group, and 12 in the control group. Demographically, they were randomly and equally assigned to each group. All the patients who completed their follow-up were observed for six months. The mean age at the time of the procedure was 48 years (range 23–93); 15 (50%) individuals were female, and 27 (90%) individuals were Hispanic. Six individuals (20%) underwent PK due to a previous failed PK, eight (27%) due to corneal decompensation or scarring secondary to trauma, four (13%) due to persistent bullous keratopathy, and 12 (40%) due to ectasia. The demographic characteristics are presented in Table 1. Donor tissue characteristics are described in Table 2.

**Table 1:** Demographic characteristics.

	Mean	Median	Range	SD	Controls	aPRP	PRGF-Endoret
Age (years)	48	45	23–93	18	53	43	44
			*n*	%	*n*	*n*	*n*
Ethnicity	Hispanic		27	90*	9	7	11
	Caucasian		3	10	2	1	0
Gender	Female		15	50	5	3	7
	Male		15	50	6	5	4
Eye	Right eye		15	50	7	4	3
	Left eye		15	50	4	4	8
PK indication	Ectasia		12	40	3	4	5
	Trauma		8	27	4	1	3
	Repeat PK		6	20	3	1	2
	PBK		4	13	1	2	1
Combined surgery	Triple procedure		3	10	1	1	1
	Secondary IOL		3	10	1	1	1
	Silicon oil removal		2	7	1	0	1
Subsequent surgeries	CEIOL		1	3	1	0	0
	Secondary IOL		1	3	0	0	1
	Dehisced PK		2	7	1	0	1
Intraocular pressure	Low (<8 mmHg)		1	3	0	0	1
	Normal (8-21 mmHg)		26	87	10	6	10
	High (>21 mmHg)		3	10	1	2	0

*p < 0.05. PK, penetrating keratoplasty; PBK, persistent bullous keratopathy; IOL, intraocular lens; CEIOL, cataract extraction with IOL implantation. Subsequent surgeries = procedures in the first 6 months of follow-up after PK. intraocular pressure taken on post-operative day 7 with Goldmann applanation tonometry.

**Table 2:** Tissue characteristics.

		Mean	SD	*p*-value
Preservation time (days)	Controls	3.9	0.7	
	APP	3.5	0.5	0.6
	aPRP	4.1	0.6	0.4
	PRGF- Endoret	2.5	0.6	0.1
Tissue ECD	Controls	3,227	214.8	
	APP	3,234	264.1	0.4
	aPRP	3,180	272.2	0.3
	PRGF- Endoret	3,278	263.5	0.2

ECD, endothelial cell density; SD, standard deviation; APP, autologous plasma products; aPRP, activated platelet rich plasma; PRGF-Endoret, plasma rich in growth factors.

### Postoperative outcomes

**Figure 3:**
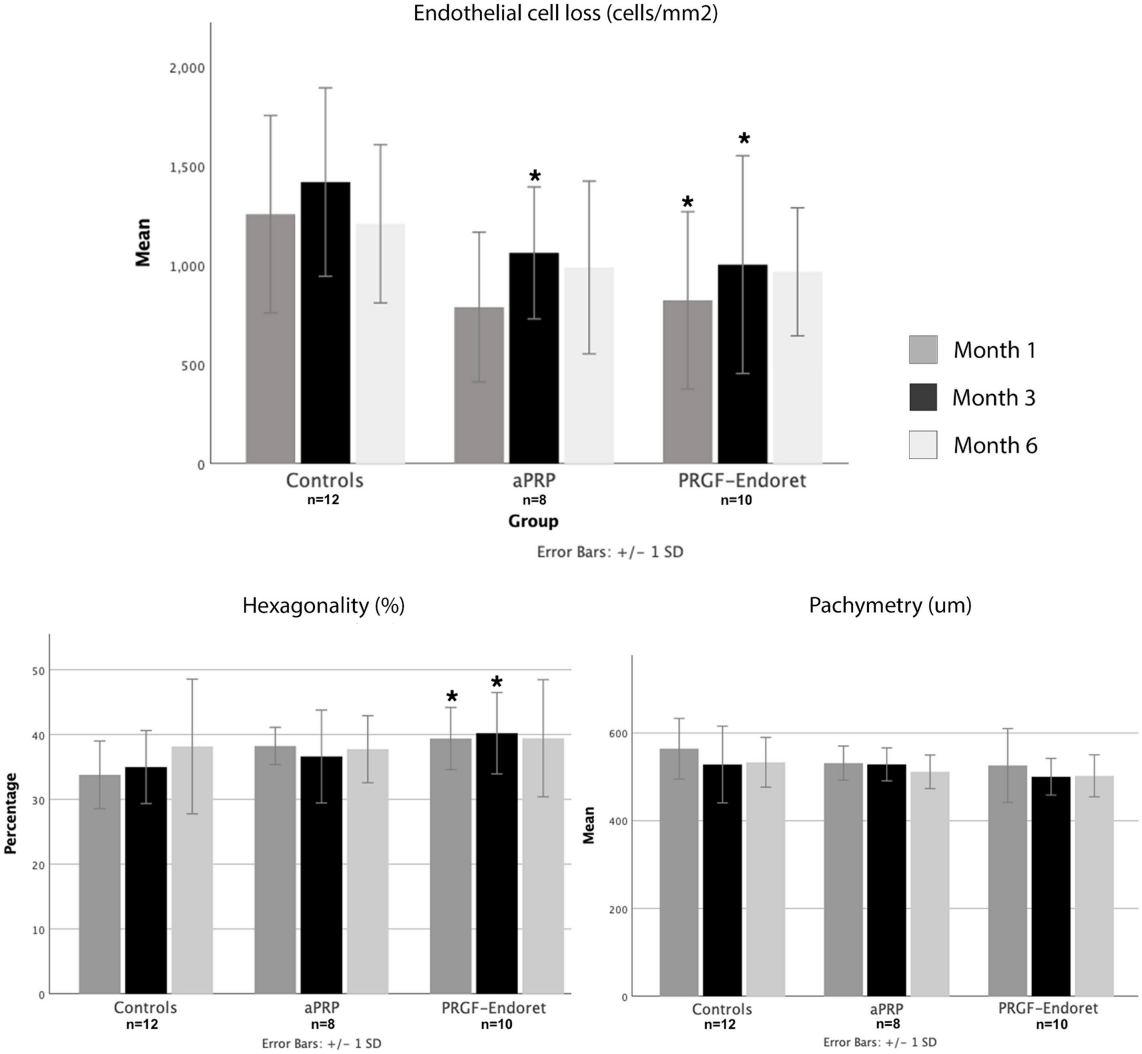
Comparison of mean endothelial cell loss **(A)**, hexagonality **(B)** and pachymetry **(C)** between groups at 1, 3 and 6 postoperative months. *Significant difference from controls at the same time point (month).

None of the patients had serious adverse events or an abnormal postoperative course related to aPRP or PRGF-Endoret treatment. In the first postoperative month, the control group presented a significantly higher mean ECL (1,199 cells; 37% loss) compared to patients who underwent incubation with autologous plasma products (APP) (806 cells; 25% loss; *p* = 0.02) (Figure 3; Table 3). After stratifying groups, a significant difference was only noted in the PRGF-Endoret group (*p* = 0.03); a significant difference was noted in both APP groups in the third month (APP 33% vs. Controls 44%, *p* = 0.02). Overall, a lower ECL in the autologous plasma-treated groups was maintained in the sixth month (APP 30% vs. Controls 36%, *p* = 0.14). Outcomes are summarized in Table 3.

**Table 3:** Outcomes.

	*n*	Month #1				Month #3				Month #6			
		ECL (cells/mm^2^)	ECL (%)	SD	*p*-value	ECL (cells/mm^2^)	ECL (%)	SD	*p*-value	ECL (cells/mm^2^)	ECL (%)	SD	*p*-value
Controls	12	1,199	37	497		1,422	44	474		1,178	36	398	
APP	18	806	25	414	**0.02**	1,059	33	446	**0.02**	979	30	373	0.11
aPRP	8	790	25	377	0.06	1,061	33	332	**0.04**	988	31	435	0.15
PRGF-Endoret	10	822	25	446	**0.03**	1,057	32	645	**0.05**	969	30	497	0.17

Pachymetry		(um)		SD	*p*-value	(um)		SD	*p*-value	(um)		SD	*p*-value
Controls	12	565		66		507		33		519		57	
APP	18	529		72	0.11	514		41	0.34	505		43	0.24
aPRP	8	531		38	0.11	528		37	0.12	511		38	0.37
PRGF- Endoret	10	526		84	0.14	500		42	0.34	498		51	0.21

Hexagonality		(%)		SD	*p*-value	(%)		SD	*p*-value	(%)		SD	*p*-value
Controls	12	33		5.6		34		6.9		38		9.8	
APP	18	39		4.2	**0.005**	38		6.7	**0.05**	40		6.9	0.36
aPRP	8	38		2.8	0.062	36		9.8	0.2	38		5.1	0.46
PRGF-Endoret	10	39		4.78	**0.009**	40		6.2	**0.03**	41		8.6	0.25

ECD, endothelial cell density; ECL, endothelial cell loss; SD, standard deviation; APP, autologous plasma products; aPRP, activated platelet rich plasma; PRGF-Endoret, plasma rich in growth factors. Bold value denotes significance.

### Corneal thickness and endothelial cell quality

Plasma blood products reduced corneal edema faster than controls. There were no significant differences in corneal thickness between groups in the first, third, and sixth postoperative months. Regarding endothelial cell quality based on specular microscopy parameters, hexagonality was significantly better in the first and third month for patients who underwent incubation (1st 39% vs. 33%, *p* = 0.005. 3rd 38% vs. 34% *p* = 0.05). This trend was consistent through the six months of follow-up. After stratifying subgroups, hexagonality was significantly higher in the first and third months postoperatively in the PRGF-Endoret group than controls (*p* = 0.009 and 0.03, respectively).

### Visual acuity

No differences between groups in preoperative uncorrected and corrected distance visual acuities were noted. In general, there was a higher number of lines of improvement with correction in the APP groups (aPRP 6 lines, PRGF 9 lines) compared to the controls (5 lines). This was not significant. Final visual acuity, defined as the visual acuity at the end of the follow-up (6 months), was not statistically different between groups. A comparison of visual acuities is displayed in Table 4.

**Table 4:** Visual acuity.

	Controls		APRP		aPRP		PRGF	
	Mean (Snellen chart)	SD (number of lines)	Mean (Snellen chart)	SD (number of lines)	Mean (Snellen chart)	SD (number of lines)	Mean (Snellen chart)	SD (number of lines)
pre-UDVA	20/250	2	20/250	5	20/250	4	20/400	1
pre-CDVA	20/150	3	20/200	1	20/160	1	20/200	1
Final UDVA	20/400	1	20/200	3	20/300	2	20/160	4
Final CDVA	20/70	1	20/63	2	20/70	1	20/60	2
Lines improved (CDVA)	5		8		6		9	

SD, standard deviation; UDVA, uncorrected distance visual acuity; CDVA, corrected distance visual acuity.

### Safety

No serious adverse events were reported. One patient in the control group and 2 patients of the aPRP group (one had high IOP before the procedure) persisted with elevated IOP (>21 mmHg) on the seventh postoperative day. Both patients in the aPRP group were successfully treated with combination drops of Brimonidine 2 mg/Timolol 5 mg/Dorzolamide 20 mg twice daily for two weeks. The patient in the control group did not respond adequately to topical therapy and subsequently underwent valve implantation 8 months after PK. None of the patients in the treatment groups had complications in the early postoperative period, including seidel, fibrin in the anterior chamber, or late epithelization. One patient in the control group had to be excluded due to graft failure; in the 3rd postoperative month, it was still impossible to measure endothelial cell count due to persistent edema.

## Discussion

The future of corneal transplantation involves endothelial cell cultures and medical treatments that can potentiate the survival or regeneration of these cells (23). Rho-kinase inhibitors have gained momentum in endothelial cell transplantation as an alternative to block pathways in corneal wound healing, which would otherwise lead to endothelial cell remodeling and death (24). Despite several advances, some of these proposed solutions are costly and potentially difficult to access in underdeveloped countries.

Autologous blood products have been used in medicine for over 20 years (25). The ability to promote the production of FGF, TGBF, and VEGF explains their success in regenerating tissues like the corneal epithelium (20). We have previously demonstrated that after a short 15-min incubation, plasma rich in growth factors promotes salvaging pathways such as AKT and PiKT3 and blocks apoptotic proteins such as BAD and CASP3, thereby protecting endothelial cells from oxidative stress (21). The use of FGF-1 in Descemet stripping only (DSO) for treating Fuchs endothelial dystrophy has been previously explored and has shown outstanding results in clearing corneas. Specifically, FGF promotes the migration and proliferation of CECs (26, 27). Although growth factors are found in all autologous blood products, PRGF-Endoret has the highest content (28). Preparing PRGF-Endoret guarantees a controlled environment, thus ensuring higher concentrations of growth factors. After a 45-min activation with calcium chloride, the centrifuged blood can reach an increased range of growth factors, and this might explain the differences seen between the aPRP and PRGF-Endoret groups in our study. Regardless, in our patients, higher endothelial cell counts were seen in both autologous plasma product groups compared to the control group.

In our study, we saw overall better pachymetry in the first postoperative month in patients who had their corneal grafts incubated in autologous blood products, and this was consistent throughout the following months but was not statistically significant. As a somewhat subjective observation, more transparent corneas were also noted clinically by the surgeons in the first postoperative days; this can be expected as autologous plasma products knowingly benefit epithelium healing as well. Corneal edema after corneal transplantation highly influences the visual rehabilitation of the patient. We noticed that patients in the APP groups had more lines of improved corrected distance visual acuity; this was not statistically significant. Even though these initial results are encouraging, these values must be considered cautiously, as most sutures were still present at 6 months following penetrating keratoplasty, causing high astigmatism, sometimes too high to correct. We hope future studies with longer follow-up times can assess visual acuity months after suture removal.

Hexagonality is a controversial parameter reported on specular microscopy, and it is still up for discussion if this value is a helpful fact about endothelial cell quality (29). Notably, patients with Fuchs endothelial dystrophy experience pleomorphism of endothelial cells, and hexagonality is lost. Higher percentages of hexagonality (60%) are characteristic of a healthy endothelium (30). In our patients, a significant difference in hexagonality was reported, especially in the PRGF-Endoret group, where a significantly higher hexagonality percentage was noted in months 1 and 3. This might indicate the potential of PRGF-Endoret to protect corneal endothelial cells throughout the most critical months following corneal transplantation.

In our study, both plasma products proved to be safe. No serious side effects were noted in any of our patients. Intracameral PRGF-Endoret injection has previously shown a transient intraocular pressure spike (28 mmHg) in one patient that spontaneously resolved in the first four hours (31). There have been no side effects reported with intravitreal PRGF-Endoret (17). The main concerns about these blood products are (1) the possibility of acting as a growth factor for bacteria and (2) a fibrinoid reaction. Previous studies have demonstrated the bacteriostatic effect of PRGF-Endoret eyedrops in species such *Staphylococcus aureus* and *S. epidermidis* (32). Moreover, although the preparation processes for both plasma products are manual, we should consider that aPRP has a higher risk of having a fibrinoid reaction as the extraction process is less standardized (33). The step that should not be overlooked during aPRP preparation is the extraction of the plasma column, as the operator can also extract cells from the thin underlying white blood cell column; this mix-up could cause an inflammatory reaction in the anterior chamber and as some studies have pointed, decrease the clot stability and delay cell remodeling (34).

One of the patients in the control group had graft failure. This was likely due to predisposing factors like the trauma that necessitated the PK, which led to significant inflammation and the subsequent development of anterior and posterior synechiae, making successful graft integration more difficult. On the other hand, a comparable group of patients underwent PK due to trauma in the APP group, none of whom had synechiae formation or graft failure. Even with a small cohort, we suggest a potential for incorporating growth factors from autologous plasma products (APP) such as aPRP and PRGF-Endoret, which seems promising in reducing graft failure risk. These growth factors help by mitigating oxidative stress, promoting cellular repair, and reducing inflammation, collectively supporting better endothelial cell survival and function (21). The benefit of this adjuvant therapy in corneal transplantation has global implications, as preserving corneal endothelial cells can potentially increase the transplants’ longevity, decreasing the need for repeat transplants. Ensuring longer graft survival can be crucial in countries with limited tissue availability.

Our study was performed in Colombia and approved by the Ethics committee after carefully considering safety data regarding the intraocular use of APP. In terms of regulation in the United States for this type of product, it has to be clear that the use of intraocular APP is “off-label.” This is also true for using aPRP in other subspecialties, such as maxillofacial surgery and orthopedia (35). Even though PRP might fall under the umbrella of biologic drugs, it does not fit the definition of Human Cells, Tissues, and Cell and Tissue-Based Products (HCT/Ps) outlined in Title 21 of The United States Code of Federal Regulations Part 1271. Instead, its regulation pertains more to the devices used in its manufacture, with various PRP systems cleared through the 510(k) pathway, which approves products highly similar to a previously cleared device, known as a predicate device.

The results of our study should be considered in light of its limitations, such as short follow-up time and small sample size. Due to the pilot nature of this study and the limited number of participants, we acknowledge that the statistical power is reduced. The lack of tissue is a significant limitation in South America, which explains the small sample size. This study aims to provide preliminary insights and establish a foundation for larger, more definitive studies. Even though we observed a trend in the sixth postoperative month towards better quality and quantity of CECs findings should be interpreted with caution and validated in future research with larger sample sizes. The research’s small sample size and exploratory nature justify using these statistical methods; this also led to adding a wide range of patients, including patients undergoing different procedures such as cataract surgery and silicon oil removal; this might influence the rate of endothelial cell loss. Variability in pachymetry from tissue retrieval, preservation, and implantation led us to exclude tissue pachymetry values, as this would have implied manipulating the tissue before implantation for corneal thickness measurements. Donor tissue hexagonality values were unavailable for baseline comparison; this limits our ability to assess endothelial cell morphology changes objectively following the intervention.

In conclusion, our study found that aPRP and PRGF-Endoret were safe for the corneal endothelium. Based on the postoperative clinical findings, it is essential to note the non-inferiority of autologous plasma products in corneal transplantation and its potential to protect from corneal endothelial cell loss. In the future, more extensive studies are required to assess further the long-term clinical impact of intraoperative autologous plasma therapy.

## Data availability statement

The raw data supporting the conclusions of this article will be made available by the authors, without undue reservation.

## Ethics statement

The studies involving humans were approved by Escuela Superior de Oftalmología-Instituto Barraquer de América Ethics committee. The studies were conducted in accordance with the local legislation and institutional requirements. The participants provided their written informed consent to participate in this study.

## Author contributions

CM: Conceptualization, Funding acquisition, Investigation, Methodology, Project administration, Resources, Supervision, Visualization, Writing – original draft, Writing – review & editing, Data curation, Formal analysis, Software, Validation. CH: Methodology, Project administration, Supervision, Writing – original draft, Investigation, Software. CL-R: Data curation, Formal analysis, Investigation, Validation, Writing – review & editing. BS-C: Conceptualization, Funding acquisition, Resources, Supervision, Writing – review & editing. JB: Investigation, Project administration, Resources, Supervision, Writing – review & editing. AG: Conceptualization, Investigation, Methodology, Project administration, Resources, Supervision, Writing – review & editing. EO: Conceptualization, Formal analysis, Funding acquisition, Investigation, Methodology, Project administration, Resources, Software, Supervision, Validation, Visualization, Writing – original draft, Writing – review & editing. AS: Conceptualization, Formal analysis, Funding acquisition, Investigation, Methodology, Project administration, Resources, Software, Supervision, Validation, Visualization, Writing – original draft, Writing – review & editing. EA: Writing – review & editing, Supervision, Investigation, Funding acquisition.
